# Coping with and management of COVID-19 restrictions within the secure and forensic inpatient setting - a patients' and carers’ perspective

**DOI:** 10.1192/bjo.2021.814

**Published:** 2021-06-18

**Authors:** Syed Ali, Katie Glennon

**Affiliations:** 1 Sussex Partnership NHS Foundation Trust; 2 Sussex Partnership NHS Foundation Trust (SPFT)

## Abstract

**Aims:**

To seek patients' feedback on their wellbeing and the service adaptations during the COVID-19 pandemic

To obtain carers’ views on service adaptations during the COVID-19 pandemic.

To establish impact on patients' wellbeing and progress in the context of COVID-19

**Background:**

The COVID-19 pandemic resulted in unprecedented challenges faced by healthcare systems worldwide. Public Health England (PHE) provided guidance to manage the spread of the virus. In response to the national lockdown, the Forensic Healthcare Service part of Sussex Partnership NHS Foundation Trust (SPFT) took measures that were considered necessary to prevent the risk of spread to patients and staff.

Restrictions necessary to contain the virus included immediate suspension of all patients leave except emergency leave, suspension of visits by family members and professionals including legal visits and restrictions on multidisciplinary (MDT) members physically present on the wards. It was necessary to adapt our existing model of care to reflect and represent the challenges faced by such restrictions.

A service evaluation project was undertaken to ascertain the patients' and carers’ perspectives of the management of restrictions.

**Method:**

Standards

It is noteworthy that no service standards in the context of this unique global pandemic were available internationally, nationally or regionally at the time of undertaking the project.

Methodology / Data collection

An anonymous patient feedback questionnaire was developed to collect data on voluntary basis from all the inpatients within the secure and forensic CDS. Patients' feedback was broadly divided in to three sections 1) personal factors, 2) satisfaction with access to information and 3) satisfaction with services to include mental and physical well-being.

Patients' feedback was collected during a 6-week period. For observation purposes, risk comparison anonymous data were also collected. Informal Carers' feedback was collected with regard to virtual visits.

**Result:**

During the data collection period 99 out of 105 beds were occupied. The response rate was 49% (49 responders).

Overall 73% of responders expressed that their mental health was affected. Approximately 51% of responders expressed that progress towards their discharge was very much affected. 91% of responders were not coping well with the new circumstances

Overall, carers' feedback was positive in regard to provision of virtual visits.

**Conclusion:**

Our survey has shown that the necessary COVID-19 pandemic restrictions have in some domains resulted in a negative impact on patients’ mental wellbeing and progression. However, it also identifies positive areas of new practice, which have been maintained by the service.

